# Youths’ perceptions of community health workers’ delivery of family planning services: a cross-sectional, mixed-methods study in Nakaseke District, Uganda

**DOI:** 10.1186/s12889-021-10695-y

**Published:** 2021-04-07

**Authors:** Robert Kalyesubula, Jessica Mitter Pardo, Stephanie Yeh, Richard Munana, Ivan Weswa, Joseph Adducci, Faith Nassali, Mennen Tefferi, John Mundaka, Sahai Burrowes

**Affiliations:** 1 African Community Center for Social Sustainability, Nakaseke, Uganda; 2 Makerere University College of Health Sciences, Kampala, Uganda; 3 Touro University California, California, USA

**Keywords:** Adolescents, Family planning, Contraception, Reproductive health, Community health workers, Uganda

## Abstract

**Background:**

High rates of unintended adolescent pregnancy are a significant health problem in Uganda. To improve access to family planning (FP) services, community-based Village Health Teams (VHTs) are widely employed in Uganda to deliver education and services. However, evaluations of FP programs suggest that mainly older, married women use VHT FP services.

**Methods:**

To better understand youth reluctance to use VHTs, we collected quantitative FP and contraceptive-seeking behavior data from a survey of 250 youths aged 15–25 in randomly selected households in Nakaseke District, which we triangulated with data from 3 focus group discussions (FGDs) (*n* = 15).

**Results:**

Most respondents received FP services from the formal health sector, not VHTs. Only half had talked to a VHT, but 65% knew that VHTs provide free FP services, and most (82%) felt comfortable talking to VHTs about FP. The main reasons for discomfort were fear that VHTs would violate privacy (mentioned by 60% of those not comfortable), that VHTs would talk to parents (33%), shyness (mentioned by 42% of those ≤18), and fear of being judged (14%). Concern about side effects was the most common reason for not using FP methods. Survey respondents said having VHTs of the same sex was important, particularly those in the youngest age group (OR = 4.45; 95%CI: 1.24, 16.00) and those who were unmarried (OR = 5.02; 95%CI: 2.42, 10.39). However, FGD participants (who were older than survey respondents on average) often preferred older VHTs of the opposite sex, whom they viewed as more professional and trustworthy. Respondents said the primary deciding factors for using VHTs were whether privacy would be respected, the proximity of care, and the respectfulness of care.

**Conclusions:**

VHTs are a known source of FP services but not widely used by youth due to privacy and quality of care concerns. VHT messaging and training should increase focus on ensuring privacy, protecting confidentiality, providing respectful care, and addressing concerns about contraceptive side effects. Preferences for VHTs of similar age and sex may be more important for younger adolescents than older youths for whom quality concerns predominate.

**Supplementary Information:**

The online version contains supplementary material available at 10.1186/s12889-021-10695-y.

## Background

Although the Ugandan government has made impressive strides in increasing access to sexual and reproductive health (SRH) services for youth over the past 20 years, the unmet need for family planning services remains persistently high. Data from the most recent Uganda Demographic and Health Survey (UDHS) reveals that in 2016, only 10% of all women aged 15–19 and 43% of sexually active unmarried women in this age group were using a contraceptive method [1]. A quarter of women aged 15–19 had already begun having children [1]. Almost half (45%) of sexually active unmarried women aged 15–19 reported an unmet need for contraception in the UDHS. More recent data from the Guttmacher Institute suggest that the percentage of women with unmet need in this age group may be higher than 60% [1, 2]. Lack of access to family planning services has been particularly acute in Uganda’s rural areas, where the actual fertility rate is 1.3 children higher than the wanted fertility rate, and 1 in 4 females become pregnant between ages 15 and 19 [1].

The negative impact of adolescent and unwanted pregnancy should not be underestimated, as pregnancy and childbirth-related complications are a leading cause of death for females aged 15 to 19 in low-income settings [2, 3]. Pregnant adolescents experience a significantly higher rate of severe neonatal conditions such as preterm delivery, low-birthweight infants, and stillbirth. They also face an increased risk of perinatal systemic infections, eclampsia, and endometritis [1, 3, 4]. In addition, adolescent pregnancy has many economic consequences, including lower educational accomplishment and subsequent reduced vocational opportunities and financial earnings, as well as significant psychosocial consequences such as estrangement from family, depression, and even violence [5–9]. Lack of access to SRH services can also be seen as a violation of sexual and reproductive health rights, which include the right to “decide whether, when and by what means to have a child or children, and how many children to have” [10].

Using modern family planning (FP) methods reduces adolescent pregnancy and its subsequent adverse health and socioeconomic outcomes; however, willingness and ability to access SRH services may be particularly difficult for young populations. Several general barriers to accessing FP services are prevalent in low- and middle-income countries, including long distances to health facilities, high cost, inadequate supplies, and lack of choice in FP methods [11–13]. These barriers may be higher for youth and adolescents, who also face additional obstacles to care, such as provider refusal to deliver care and legal restrictions such as age of consent laws and requirements for spousal or parental permission for certain services [14–16]. In addition to these health systems and policy-level barriers to access, young women in Uganda also face significant social and cultural barriers, including sexual coercion, reproductive coercion, and gender-based violence, which are prevalent in the country [17–19]. Although the evidence is mixed, these forms of violence and coercion severely limit young women’s reproductive autonomy and may prevent them from seeking FP services and constrain their choice of FP methods [20–23].

In addition to problems with access, acceptability and demand for services may also be low in young populations due to concerns about privacy, autonomy, and disapproving and judgmental providers [24–26]. Large-scale population-based surveys among youth in Kenya and Zimbabwe, for example, have identified that, with regards to SRH services, adolescents cite confidentiality, short wait time, and low cost as important factors in determining their use of FP services [27].

To address these concerns and to encourage the utilization of services, global policymakers have promoted the creation of “youth-friendly” SRH services that have special features such as separate clinic hours and waiting areas for youth [28–31]. However, there remains a relative dearth of such youth-friendly services in Uganda and elsewhere in East Africa. While Uganda’s policies allow FP services to be provided without spousal consent or parental consent for adolescents, the policies require prior education on FP method choices [32]. Providing this education to youth has been complicated by the country’s recent switch to abstinence-only sexual health education in the public school system [32, 33].

Largely absent from policy discussions about expanding youth-friendly FP services is an analysis of how this approach aligns with another strategy commonly employed by health systems in low- and middle-income countries to increase access to, and utilization of, comprehensive FP services, namely the employment of community-based health workers (CHWs) to deliver FP education and services directly to women in their communities. Such “task-sharing” CHW-based initiatives have been credited with facilitating increases in contraception uptake in low- and middle-income countries and have been central in countries that have had dramatic improvements in FP uptake, such as Ethiopia, Malawi, and Rwanda [34–39]. In Uganda, the Ministry of Health organized CHWs into Village Health Teams (VHTs) in 2001, and since that time, CHWs have commonly been referred to as VHTs. VHTs are volunteers elected by their communities, who act as the first point of contact with the health system at the household level. They provide health education, case management of common health conditions, distribution of medicines and supplies, and referral and follow-up services for health facilities. The Ministry requires that VHTs be over the age of 18, and most programs that use VHTs also require that they be literate in the local language [40]. In 2011 VHTs were authorized to directly provide SRH services to the public, including delivering an array of contraceptive methods such as condoms, oral contraceptives, emergency contraceptives, and injectables [41, 42]. Since that time, there has been little reporting on how well VHTs have performed at meeting the needs of younger community members for FP services. Outside of Uganda, one of the few CHW FP studies that focused on young women found evidence of CHW reluctance to provide services to young, married women who had not yet had children. The authors speculate that this reluctance may have been due to a cultural expectation that young women “prove their fertility” early in marriage before spacing births [38].

Our study’s motivation is a program led by a Ugandan non-governmental organization, ACCESS-Uganda (African Community Center for Social Sustainability), in which VHTs provide community members with health education and contraceptive supplies and make referrals to primary care health centers for long-acting and permanent contraceptive methods (LAPM). Internal evaluations of the program found that beneficiaries were primarily older and married women or women who had already given birth, rather than younger, unmarried women or nulliparous but sexually active women. We, therefore, undertook this study to investigate how youth in the project area perceived VHT outreach services and to identify barriers to youth utilization of these services. We were particularly concerned to learn about youths’ desire for younger, peer VHTs, as community-based family planning programs frequently exclude adolescents from serving as CHWs [38]. Understanding how youths seeking FP services perceive the approachability of CHWs like Uganda’s VHTs is crucial for planning and managing the growing number of these initiatives. Such information is particularly crucial for programs operating in rural areas such as our study site, where CHWs may be the only easily accessible sources of comprehensive FP education and services.

## Methods

### Study design

The study used a mixed-method, cross-sectional design. The primary approach was quantitative, employing survey data from randomly selected households, with qualitative focus group data used to inform and explain survey results.

### Study setting

We conducted the study in Nakaseke District. Nakaseke District is a largely rural area located approximately 42 miles from Kampala. The district consists of 15 sub-counties, which are further divided into parishes, villages, and households. The district age distribution is similar to the national distribution: 18% of the population of nearly 200,000 people are between the ages of 15–24 [43]. Approximately 18% of young women between 12 and 19 years of age have already given birth. At the time of the study, The ACCESS-Uganda Family Planning program operated in 6 of 15 sub-counties with 98 VHTs estimated to cover approximately 119,339 community members [44].

### Participant selection

One parish was randomly selected from each of the six sub-counties in the ACCESS-Uganda Family Planning program. The number of participants recruited per sub-county was proportional to the youth population size in the sub-county, based on the 2014 National Population and Housing Census [43]. We calculated a sample size of 229 individuals based on Cochran’s formula, with a 95% confidence interval and a 5% margin of error. We then rounded up the sample size to 250 individuals to account for possible non-response.

We used a random-walk technique for household selection. On arriving at the selected parish, the data collection team continued to travel on the main road leading away from the center. If there were several roads, one was picked at random using a series of coin flips. The data collection team then stopped in at every third village along the road for sampling. The team walked to the center of the village, asking directions if necessary, and then spun a pencil to determine the direction they would travel. They then recruited participants from every third household in that direction, returning to the center to repeat the random walk as necessary. If a selected household did not have persons in the target population available for participation, the data collection team proceeded to the next household for sampling. All youth aged 15–24 who lived in the household were eligible for inclusion in the study, but only one youth per household was selected for the survey on a first-come-first-served basis.

Participants were asked if they were willing to be contacted for participation in a focus group discussion (FGD) during the consent process. We conducted 3 FGDs (*n* = 15) with respondents purposively selected from the list of willing survey participants to ensure a mix of different sexes, age, parity, and educational status. FGDs were organized by sex into a female, male, and mixed-sex group.

### Ethics and approvals

The study was approved by the Institutional Review Boards at Touro University California (#PH-0518) and the Mulago Hospital Research and Ethics Committee (#1583). All participants were told the aims of the study, that their participation was voluntary, and that they could withdraw participation at any time without consequences. In addition, written consent or assent for participation was obtained from all participants. Parental permission was also obtained for all respondents under the age of 18. The procedure for obtaining permission from parents and guardians for minors who aggreged to participate in the study was approved by the Institutional Review Boards/Ethics review committees that reviewed the study protocol.

### Data collection

Data collection took place in February and March 2019. The survey instrument and focus group guide were developed by adapting items found in internationally recommended instruments [45] and by reviewing the literature for East Africa-specific constructs to incorporate into the adaptation [27, 46–48]. The instrument contained questions on demographics, sexual and care-seeking behavior, barriers to contraceptive use, and knowledge of, and attitudes towards, VHT FP services offered. Both tools were developed in English and then translated into Luganda, the local language for administration (see Supplemental File 1). The surveys contained 32 close-ended questions. Before data collection, the survey was pre-tested with 15 youth to obtain feedback on the difficulty of use, readability, and interpretability.

The survey was interviewer-administered using a paper instrument. Trained students from ACCESS’ Health Training Institute of Nursing and Midwifery collected survey data after completing a one-and-a-half-day training on the study’s goals, randomization and survey procedures, and research ethics. A Touro University California master’s student coordinated data collection under the supervision of ACCESS Uganda’s Family Planning program staff and project Principal Investigators. The data collection team and coordinator debriefed after each day in the field to discuss problems and plan the next day’s activities. Every evening, the project coordinator entered the survey data into a secure online spreadsheet that the project investigators reviewed, allowing them to flag data quality problems to address in the debriefing sessions.

We conducted three FGDs after the surveys were completed in order to clarify and explain survey findings. The semi-structured focus group guide was revised based on questions that emerged from the preliminary analysis of the survey data. A professional facilitator from the ACCESS team conducted the FGDs in a conversation-like manner, in Luganda, following the guide. The FGDs were held at the ACCESS-Uganda conference room or ACCESS-affiliated health centers after hours. Each took approximately 60 min to complete and was audio-recorded with informed consent. The project coordinator participated in all three focus group discussions, recording notable comments, documenting participant behavior, and following up on facilitator questions.

### Data analysis

Survey data were entered into a Microsoft Excel spreadsheet, then exported to Stata (version 16) for analysis. We produced descriptive statistics on participant demographics, sexual history, and family planning information and services sources. We also conducted bivariate and multivariate logistic regression to determine factors associated with comfort interacting with VHTs for FP services.

FGD audio transcripts were transcribed verbatim and then translated into English and analyzed thematically. FGD facilitators conducted a brief preliminary analysis after each FGD to identify critical issues to be explored or clarified in subsequent FGDs. The transcripts were read through several times: first by a research assistant (MT) and a project investigator (SB) to develop a codebook of themes in an iterative process based on both the a priori research questions and themes emerging from the data. A second research assistant-investigator team (JMP and SB) then hand-coded paper transcripts and extracted key illustrative quotes to illuminate the themes.

## Results

### Sample characteristics

All 250 sampled households that reported having a youth eligible for inclusion agreed to participate in the study for a reported 100% response rate. We note, however, that households that did not want to participate may have stated that they did not have eligible household members rather than directly refusing to participate. The sample was relatively old, with only 21% of respondents aged 18 years or younger and slightly more than one-third over the age of 21 (see Table 1). The mean age was 20.4. Most respondents (72%) were female, and slightly more than half (54%) had completed secondary school reflecting the sample’s age structure. Almost all (81%) were not currently in school. Approximately a third of respondents (29%) were married. Forty-three percent had children, including 31% of those ≤18 and 29% of those 19–21 years old. The ≤18 age group had had 1.5 children on average.

**Table 1:** Sample Characteristics of Youths from Nakaseke District, Uganda

	Number (***n*** = 250)	Percent
Sex (Female)	178	71%
Age Group		
18 Years of Age or Younger	54	22%
19–21 Years Old	111	45%
Older than 21 Years of Age	82	33%
Highest Level of Education		
Primary	96	38%
Secondary	136	54%
Tertiary	10	4%
None	8	3%
Currently Attending School (No)	203	81%
Marital Status		
Single	128	51%
Unmarried in Relationship	44	18%
Married	72	29%
Divorced/Widowed	6	2%
Have had Children	98	43%

### Sources of family planning education

Almost all respondents had heard of the term “family planning” at some point in their lives (see Table 2). However, only 63% had received formal FP education. Of the respondents who had received FP education, 72% received it from formal healthcare facilities, either at hospitals or community clinics. Only 20% had received FP education from VHTs.

**Table 2:** Sources of Family Planning Education Among Youth in Nakaseke District, Uganda

	Number (n = 250)	Percent
Ever Heard the Term “Family Planning” (Yes)	236	94%
Ever Received FP Education (Yes)	152	63%
Where Received FP Education (*n* = 152)		
Hospital	94	62%
Community Clinic	15	10%
Village Health Team	31	20%
Other	12	8%

### Sexual history and family planning use

Almost all respondents reported having had a sexual encounter (90% of all respondents and 70% of those age 18 and under). A third of the sample reported having had sex by age 15, and the majority (62%) had their first sexual encounter between the ages of 15–19 (See Table 3). Most (65%) were currently having sex, including 47% of those ≤18. Although most respondents (72%) had used an FP method in the past, only 55% of those age 18 and under-reported having ever used an FP method. Most said that they would go to a hospital (67%) or a health center (24%) if they needed FP services. Only 9% said that they would go to a VHT.

**Table 3:** Sexual History and Family Planning Use among Youths in Nakaseke District, Uganda

	Number (***n*** = 250)	Percent
Ever Had Sex (Yes)	226	90%
Age of First Sexual Encounter		
Less than 15 Years of Age	68	31%
15–19 Years Old	134	61%
20–25 Years of Age	16	7%
Currently Having Sex (Yes)	146	65%
Ever used Family Planning (Yes)	162	72%
Where would go for Family Planning Services (*n* = 239)		
Hospital	160	67%
Community Clinic	58	24%
Village Health Team	21	9%

**Fig. 1:**
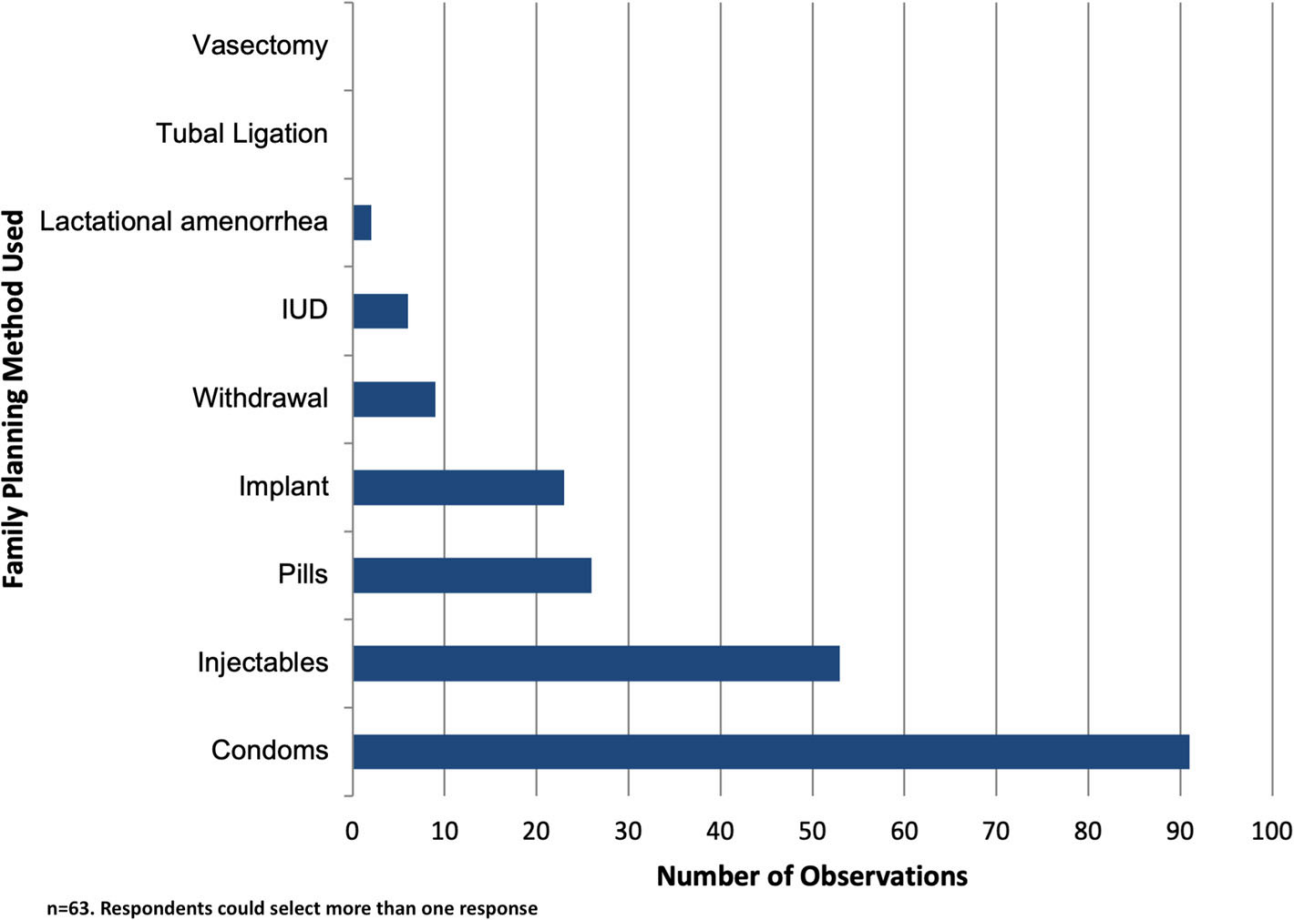
Family Planning Methods Used by Youths in Nakaseke District, Uganda (*n* = 162) ‡ Respondents could select more than one response

Condoms were the most common family planning method used (see Fig. 1), with injectables and oral hormonal contraceptives the second and third most frequently mentioned. Condom use was significantly higher among men and unmarried respondents. Other LAPMs such as intrauterine devices and implants were rarely mentioned. LAPMs use was particularly low in the youngest age group, who reported the highest proportion of injectable use and the lowest proportion of implant use.

Having had children and being sexually active were significantly and positively associated with ever having used FP when controlling for other socio-demographic factors: AOR = 3.205; (95%CI: 1.229, 8.356) and AOR = 5.252 (95%CI: 2.41, 11.41) respectively (see Table 4).

**Table 4:** Logistic Regression: Factors Associated with Ever Using Family Planning (*n* = 221)^‡^

Factor	Adjusted Odds Ratio	95% Confidence Interval
Female	0.493	[0.219,1.109]
Age Group		
Less than 18 Years Old	0.726	[0.273,1.929]
19–21 Years Old	1.315	[0.570,3.033]
Older than 21 Years Old	Base	
Educational Level		
Primary	Base	
Secondary	1.848	[0.877,3.892]
Tertiary	No observation	
None	1.554	[0.231,10.45]
Marital Status		
Single or Unmarried	Base	
Married or Divorced	0.405	[0.151,1.082]
**Have had Children**	3.205*	**[1.229,8.356]**
**Currently Having Sex**	5.252***	**[2.417,11.41]**

* *p* < 0.05, ** *p* < 0.01, ***p < 0.001

‡29 observations were dropped from the regression due to missing responses.

### Health seeking behavior and barriers to care

Almost all respondents (96%) said that they knew where they would seek FP services if they needed them. When questioned about whom they would ask if they had a question about family planning, most said they would seek a health professional (66% overall, 60% in the youngest age group). The next most frequently mentioned potential sources of information were VHTs (27%) and friends (21%). There were no significant differences by age group or marital status in preferred sources of FP information.

**Fig. 2:**
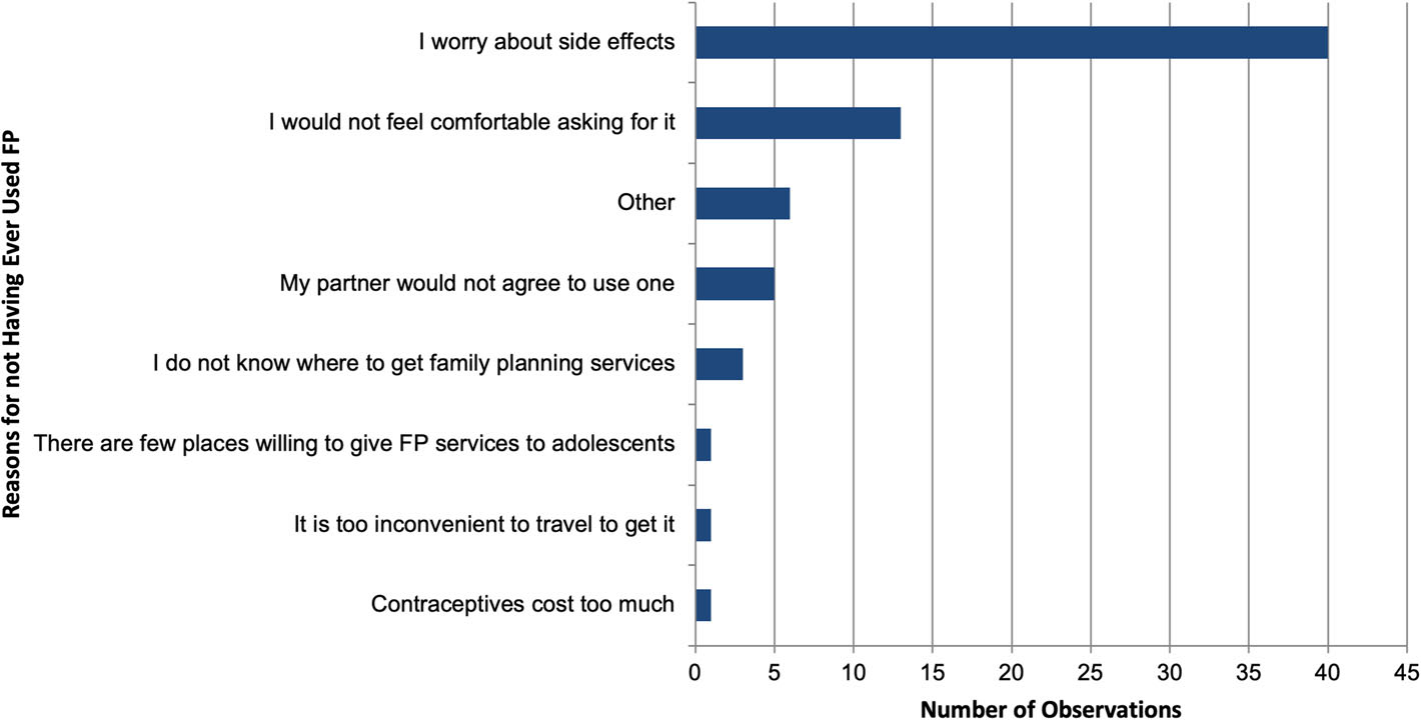
Reasons for not Having Ever Used Family Planning Services in Nakaseke District, Uganda (*n* = 63) ^**‡**^**.** ‡ Respondents could select more than one response

Barriers to accessing FP services identified in FGDs included lack of time, distance to services, and cost. In the survey, the most common reason for not using FP was concern about potential side effects (see Fig. 2). Participants in all three focus groups also reported concerns and misconceptions about potential side effects such as amenorrhea, congenital disabilities, and infertility. In focus groups, concerns about side effects were more pronounced among women than men.

“I used an injection before and spent a full year without getting my menses, but when they resumed, they were normal, but I fear to do the injection again because I was told I would get fibroids or fail to conceive. That scared me.” – (Female Respondent, FG2)

However, the most consistent theme, cited most frequently across all three groups when discussing barriers, was the stigma surrounding family planning services and the resulting fear and shame that accompanied attempts to access these services. For example, respondents often stated that the use of family planning services by unmarried women suggested promiscuity.

“*If your parent finds out that you are on family planning, he/she would just know that you are sleeping around with men. Otherwise, why would you be on them? […] They think you shouldn’t be having sex at that time. So if you’re not having sex, why would you go for family planning methods?” – (Female Respondent, FG1)*

“Most people know (about family planning methods), but fear accessing them. For example, I cannot walk to a shop to buy a condom even if you gave me money to [because] people that see would think that you are going into a sexual act.” – (Female Respondent, FG2)

While the theme of stigma among female respondents concerned stigma aimed at others and themselves, male respondents only discussed women being stigmatized for FP use. However, family planning services were deemed appropriate for married women, as this was associated with birth-spacing and was considered a decision to be made between a married couple.

“If you see a girl who is not married using that (long-term planning) method, you think that she is sleeping around with many men, but with a married woman that can’t come to my mind because I know that it is between her and the husband.” – (Male Respondent, FG3)

### Perceptions of VHTs

VHTs were visible to the youth in our sample: 72% of respondents said they had seen a VHT working in their community, and 65% knew that VHTs provide free FP services. However, only half of the respondents had talked to a VHT and only 38% of those ≤18 years of age. Most (84%) said they would feel comfortable talking to a VHT about FP services (see Table 5).

**Table 5:** Perceptions of VHTs Among Youths in Nakaseke District, Uganda

	Number (n = 250)	Percent
Comfortable Talking to VHT about *Health*	220	88%
Comfortable Talking to VHT about *Family Planning*	208	84%
Reason for Potential Discomfort Talking to VHT about Family Planning^**‡**^		
I would be afraid the VHT would share our conversation with my parents	55	33%
There would be no privacy when talking to VHTs	102	61%
I would not want to reveal that I am having sex	28	17%
I would feel shy talking about sex	37	22%
I would be afraid that the VHT is judging me	25	15%
I would be afraid that the VHT would not treat me well	12	7%
Important that VHT is the Same Sex	212	85%
Important that VHT is the Same Age	196	78%

‡Respondents could select more than one response

When asked why they would not feel comfortable speaking to a VHT, the main reasons given were fear that VHTs would violate privacy (mentioned by 61% of those not comfortable), that VHTs would talk to parents (mentioned by 33%), shyness (mentioned by 22 and 42% of those ≤18), and fear of being judged (mentioned by 15%). The centrality of stigma and privacy concerns found in the survey data was echoed in all three focus groups (see Table 6).

**Table 6:** Focus Group Themes of Privacy Concerns Regarding Use of VHTs

	Theme 1: Prefer someone from a different village	Theme 2: VHTs gossip	Theme 3: Young VHTs are not preferred to older
**Focus Group #1: Mixed Sexes**	**Moderator:** So you don’t want a VHT to come to your community and provide these services? **Respondent**: No. Take you for example, you are new to the village, you work and we know that you will leave and that you are not going to spread the rumors, then I go to you [to receive services].	**Respondent**: VHTs, mostly those who work in villages, after coming to understand you, they will make sure to spread the rumor.	**Moderator 1**: What if a VHT was like somebody your age, would that make it different? **Respondent**: No, they would still talk. Those are even worse. **Moderator**: So it’s better if they are older? **Respondent**: The older one will be experienced in that field and will not spread the rumor to our fellow youth.
**Focus Group #2: Females only**	**Respondent:** The one from your village will spread rumors about you because they know where your house is.	**Moderator 2**: Don’t you like VHTs? **Respondent**: They spread rumors.	**Respondent:** It can work for you if you trusted that peer. **Moderator 1**: So you don’t want to go see a VHT if they are older, right? **Respondent**: If you trust them…s/he is safe.
**Focus Group #3: Males only**	Not mentioned	**Moderator:** So do you think the VHTs in your community should be talking to boys in your community about this kind of thing? **Respondent**: They should be doing it but I for myself I can’t open up to him or her because she might discuss about me with her friends in the community.	**Respondent**: No we can’t open up to our age mates. **Moderator:** So it is better if they are older? **Respondent:** Yes.

Focus group participants consistently mentioned privacy concerns when discussing their willingness to use VHT services, specifically, the widely held belief that VHTs would gossip about their clients and “spread rumors.” These confidentiality breaches were seen as particularly acute because they would occur in one’s own village. Participants often stated that a VHT from a different village, or VHTs who were older, might be relatively more trustworthy in keeping sensitive information private (see Table 6).

Privacy concerns were also central for deciding whether or not to use VHT services in the future. Survey respondents said the main factors for decision-making about using VHTs were whether privacy would be respected (mentioned by 45% of respondents), the proximity of care (22%), and the respectfulness of care (27%). There were no significant differences in deciding factors by age group, sex, or marital status. The cost of VHT care was not a significant concern for survey respondents (mentioned by only 8%).

The privacy theme was related to a parallel theme concerning a desire for professionalism and solid training in FP service providers. For example, in the focus groups, respondents said they preferred formal, facility-based services to VHTs not only because of privacy concerns but also because of worries about VHT’s capacity and ability to provide quality FP services. Several respondents believed that VHT capacities were limited to non-family planning services and that VHT service quality was questionable.

“[VHT] services are known; mosquito nets, immunization of children. That is all they can handle: distribution of mosquito nets.” – (Sex Unrecorded, FG1)

“You get better information at the clinic than from the peer VHTs.” – (Female Respondent, FG2)

The only factor significantly associated with the level of comfort speaking to a VHT was educational level. Respondents with a tertiary level of education were much *less* likely to say that they were comfortable speaking to a VHT than those with a primary school education when controlling for other behavioral and demographic factors (AOR = 0.056, 95% CI: 0.011,0.294) (see Table 7).

**Table 7:** Logistic Regression: Factors Associated with Being Comfortable Talking to a VHT about Family Planning (*n* = 221) ‡

Factor	Adjusted Odds Ratio	95% Confidence Interval
Female	0.863	[0.310,2.401]
Age Group		
Less than 18 Years Old	1.194	[0.294,4.858]
19–21 Years Old	0.704	[0.244,2.025]
Older than 21 Years Old	Base	
Educational Level		
Primary	Base	
Secondary	0.395	[0.136,1.149]
**Tertiary**	0.056***	**[0.011,0.294]**
None	0.41	[0.040,4.216]
Marital Status		
Single or Unmarried	Base	
Married or Divorced	0.833	[0.275,2.522]
Have had Children	1.407	[0.457,4.337]
Currently Having Sex	1.848	[0.752,4.544]

* p < 0.05, ** p < 0.01, ***p < 0.001

‡29 observations were dropped from the regression due to missing responses.

When we explored the importance of VHTs’ sex and age in influencing the utilization of VHT services, we had mixed results. The majority of survey respondents (85%) said having VHTs of similar sex was important to them, particularly those in the youngest age group (OR = 4.45, 95% CI: 1.24, 16.00) and those who were single or unmarried (OR = 5.02, 95% CI: 2.42, 10.39). Similarly, most survey respondents (74%) also said that having VHTs of a similar age was important, and married respondents were significantly more likely to say so (OR = 2.84, 95% CI: 1.53, 5.29). However, several FGD participants preferred VHTs who were older than themselves because they viewed older VHTs as being more experienced, professional, and trustworthy.

“I can’t talk with my age mate because in most cases if your age mate is advising you; you might not take it seriously. So it is better if the person is older than you. If this person gives you advice, you can easily take it on because of the respect you have for him.” – (Male Respondent, FG3)

FGD participants also voiced differing, conflicting views on VHT sex preference. Some preferred same-sex VHTs; some female participants preferred male VHTs because they believed they would be less likely to gossip; and some male participants said they preferred female VHTs because they were more professional than male VHTs.

## Discussion

This study set out to assess FP health-seeking behavior among youths in Nakaseke, Uganda, and their awareness, attitudes, and practices regarding VHT provision of FP services.

We found evidence of a significant unmet need for FP services in this sample. Most respondents were sexually active, and a significant proportion of those under 18 already had children. While 70% of youth aged 18 and under-reported having had sex, only 55% reported having ever used family planning. This evidence of an unmet need for FP services echoes the findings of previous Ugandan studies [2, 13, 49]. It underscores the work that remains to be done to ensure access to, and utilization of, FP services among youth.

Several access and utilization barriers that VHTs should address, including time, cost, and distance to FP services, were still frequently mentioned as barriers to care in this community where VHTs are active. However, the most frequently mentioned reason for not using FP was concern about side effects, particularly from LAPMs. Several sub-Saharan African studies have found that concerns about side effects and misconceptions about the effects of long-term contraceptive use are common [50–54]. While these misconceptions and concerns are not uniquely found in VHT-based FP programs, the presence of VHTs in the study communities has not been sufficient to dispel them. Together the persistence of access barriers and misconceptions regarding contraceptive side effects reflect the finding that youth are not utilizing VHT services.

Overall, we found high awareness of VHTs services but little prior use and low willingness to use these services in the future. Despite the presence of VHTs in their communities, only half of the youth surveyed had ever talked to a VHT. When asked about FP education sources, only 20% mentioned VHTs as a source; however, we note that this low estimate could be an artifact of the survey instrument that did not allow respondents to select multiple FP education sources. Nevertheless, even though most survey respondents (84%) said that they would feel comfortable talking to a VHT about family planning, when asked where they would seek FP information if they needed it, only 9% mentioned a VHT as their first choice.

When we asked youth about their reasons for non-utilization of VHTs, we found that worries about privacy and confidentiality were the primary drivers of reluctance. Both survey and focus group respondents indicated that they were worried that VHTs would gossip or otherwise share information and that this would reveal their sexual activity to parents and might paint them as promiscuous in their peers’ eyes. Internalized stigma and the related privacy concerns are commonly cited as reasons for low use of facility-based FP service among adults and youth in sub-Saharan Africa [11, 55, 56], and privacy and confidentiality protections are frequently used as an indicator of the “youth friendliness” of facility-based services [57]. However, our findings suggest that these privacy concerns may be particularly acute for community-based FP programs that use CHWs such as Uganda’s VHTs.

Previous studies of HIV/AIDS and maternal and child health programs that use CHWs have found that community members worry about sharing information with CHWs because CHWs are seen as members of the community who are likely to pass on private information to peers and neighbors [58, 59]. Lack of trust of CHWs and fear of confidentiality breaches have been cited in many other CHW health programs and have been identified as reasons for low acceptance of CHW services [58]. These studies, like ours, find that community members may prefer to speak about sensitive matters to providers who live outside of the community and to those in formal health facilities, because these providers are seen as being more likely to protect confidentiality due to their distance, their professional codes of conduct, and their more extensive training [59].

Our respondents seemed not only to fear having sensitive information shared with the community, but also to fear judgment from VHTs themselves. Approximately 15% of respondents cited a fear of judgment as the reason for discomfort in talking to VHTs; and 7% said that they would fear that a VHT would not treat them well. This fear may be justified, as previous studies in Uganda have found that providers can be disapproving of adolescents engaging in premarital sex and are often reluctant to give adolescents a full range of family planning services [60].

Overall, prior studies and ours suggest that the main strength of CHWs—their rootedness in their communities and its norms—may also make community members reluctant to talk to them about stigmatized or socially sanctioned behavior such as premarital sex among adolescents. Several studies have documented that CHW attitudes and beliefs may reflect conservative norms that are prevalent in their communities and that CHWs are often embedded in local power structures [58, 61–65], and provider bias has been documented as a critical factor affecting the uptake of contraceptive services, especially among adolescent, unmarried, or nulliparous women in other studies in sub-Saharan Africa [66, 67]. In these studies, parity and marriage are often positively associated with modern family planning use [66], which is consistent with our findings that married youth face less stigma in using FP services, as family planning decisions are seen as marital decisions. Youths’ awareness of possible provider bias may partially explain the reluctance to utilize VHT FP services found in our study.

One proposed means of increasing provider trust in services targeted at youth has been using peer-providers of similar age and sex. While studies have found that having CHWs of the same sex as clients increased uptake of services [61], our study finds mixed support for this intervention. Most survey respondents said that having VHTs of similar age and sex was desirable, and having VHTs of the same sex was particularly important for younger respondents. However, many focus group participants (who were older than survey respondents) preferred VHTs who were older than them because they viewed older VHTs as more professional. Several said that they preferred providers of the opposite sex. Female focus group respondents believed that male VHTs would be more likely to keep conversations private, while some male focus group respondents thought female VHTs would be more professional. Overall, our data suggest that preference for similar sex VHTs was not unanimous and, along with age matching, may be a more important consideration for younger clients rather than older youth.

Focus group responses to questions about age and sex matching highlight another theme found in provider choice discussions, namely the importance of quality of care. Preferences for a particular sex of VHT were driven not by which sex would be the most sympathetic or relatable but rather by which was seen as being the most professional. Similarly, the main reason for not preferring VHTs of similar age was concern that younger VHTs would not be as professional and knowledgeable as older VHTs. We also see in the focus groups that VHTs may be viewed as having a limited range of knowledge and skills and that this may influence decisions about whether to use their services. Quality concerns may be more pronounced among older, married, and better-educated youth, as indicated in our finding that higher education levels were associated with lower comfort using VHT services. It could be that more educated youth perceive VHTs as providing lower-quality care reserved for lower-status community members.

Despite the concerns that this study has raised about the use of CHWs to provide FP services to youth, the CHW model still has promising potential to increase access to FP services for older community members. A systematic review of 56 studies of CHW FP programs reported that 93% of studies found that CHW FP programs effectively increase the use of modern contraception, while 83% reported an improvement in knowledge and attitudes concerning contraceptives [34]. In addition, several studies have found that CHWs can improve health outcomes for HIV/AIDS and maternal and child health programs [68, 69]. Based on these findings, strong evidence exists for promoting CHW programs to improve access to FP services overall. However, the literature also suggests that there is an ongoing need to monitor and address quality of care and confidentiality concerns in CHW programs [34].

### Programmatic implications

VHT messaging and training should focus on protecting privacy during conversations and the confidentiality of information shared during these conversations to alleviate privacy and quality concerns. More attention should also be paid to providing respectful care and addressing concerns and misconceptions about contraceptive side effects. The training that VHTs receive, the range of services they provide, and qualifications that they have should be more widely discussed in health outreach activities in order to allay concerns about the quality of care they provide and to promote an image of them as professionals. More stringent screening in selecting VHTs may also be required to recruit VHTs who are open to providing services to youth.

Greater professionalization of VHTs, including more standardized training, more consistent remuneration, and greater integration with health facility outreach efforts, might improve the delivery of care, increase trust in VHTs, and improve perceptions of their competence. More rigorous professional training and better remuneration have long been VHT requests [63]. Countries such as Ethiopia that have fully incorporated CHWs into their formal national health programs with standardized training, scopes of work, and remuneration have seen increased utilization of CHWs and improved perceptions of health service capacity [70–72]. However, increased professionalization of CHWs might also reduce their ability to counsel clients. Trust between CHWs and their clients often rests on the perception of CHWs as peers rather than external health professionals. CHWs frequently use this insider status and community membership to facilitate discussions of sensitive issues [63]. Some stakeholders fear that professionalization that requires additional training and the use of scripts and protocols would change CHWs’ educational and social status and their perceived allegiances to formal government structures, which could, in turn, decrease community trust in their services [73, 74].

In addition to changes in the training, remuneration, screening, and outreach messages of VHTs, the utilization of tools to structure patient interactions such as mobile job aids to support counseling could improve VHTs’ communication with clients and the quality of care provided, thereby improving trust [75–77]. Mobile phone applications could help VHTs adhere to service delivery protocols and improve community confidence in their competence. Such applications have been widely used in FP, HIV, and maternal and child health programs [78].

When targeting adolescents in the youngest age group, younger VHTs of appropriate sex should be hired to encourage youth service utilization. More positive and creative messaging and outreach from VHTs are also needed to reassure this youngest age-group that services will be provided without judgment and to reduce the stigma associated with these services. Integrating VHT’s FP outreach into ongoing health promotion campaigns, such as vaccination drives and micro-nutrient supplementation campaigns, might increase the number of socially acceptable venues at which adolescents can interact with VHTs and receive FP counseling. VHT partnerships with existing SRH peer-education initiatives and informal health care settings such as drug shops might also help in this regard. For example, where they exist, peer educators could link youth in need of services to VHTs, and VHTs could assist in training peer educators on different FP methods.

### Strengths and limitations

This study has several strengths and limitations. It is one of the few studies of community-based family planning programs to seek youths’ views on the role of VHTs in the provision of family planning services. Also, it uses both qualitative and quantitative data to gain a deeper understanding of their health-seeking behavior than could be obtained by either approach alone. Finally, it examines youth interactions with VHTs in a setting where VHTs have been actively providing family planning services for several years. However, this study is conducted in a single district in Uganda, limiting its generalizability to the rest of the country and the region. The focus groups were small in size and number, which did not allow us to explore in-depth how themes varied across demographic groups. The wording of several of our survey items may have limited respondents’ ability to tell us their sources of FP education and services. For example, drug shops were not included as an FP service source, and respondents could only list one source of past FP education. Additionally, data collection was conducted by staff associated with ACCESS, an organization well known in the study area; this may have led to social desirability bias in responses. If social desirability bias was a significant factor, it would suggest that VHT use and rates of reported comfort are inflated in our findings and may, in truth, be lower than reported.

Only a quarter of our survey respondents were male, and only 39% of all respondents were adolescents (between ages 10–19). Adolescents may be less comfortable talking to VHTs about family planning than older youth due to stigma, particularly in a predominantly Catholic area such as Nakaseke. This further suggests that our estimates of VHT use might be inflated.

## Conclusions

Our study finds that VHTs are a known source of FP services among older youth in Nakaseke District, but are not widely used, due primarily to privacy concerns surrounding the use of stigmatized FP services. Our findings also suggest that the perceived trustworthiness and competence of VHTs of similar age and sex may vary by age group. Further studies to compare preferences for peer VHTs in different youth age groups may be needed to guide the recruitment and training of VHTs for community-based FP programs in Uganda.

## Supplementary Information

**Additional file 1 Appendix A** Data collection instruments. The data collection tool contains the questionnaire and the guidelines for the focus group discussions (FGD)

## Data Availability

The datasets generated and analyzed during the current study are not publicly available due to a lack of Institutional Review Board authorization for public posting of data. However, they are available from the corresponding author on reasonable request. Data requests can be sent to Robert Kalyesubula (rkalyesubula@gmail.com).

## Abbreviations

ACCESS: African Community Center for Social Sustainability
CHW: Community health worker
(U)DHS: (Ugandan) Demographic and Health Surveys
FP: Family planning
HIV/AIDS: Human immunodeficiency virus, acquired immunodeficiency syndrome
LAPM: Long-acting and permanent methods
SRH: Sexual and reproductive health
VHT: Village Health Team
