# Supplementary material for: Youths’ perceptions of community health workers’ delivery of family planning services: a cross-sectional, mixed-methods study in Nakaseke District, Uganda
Source: BMC Public Health. 2021 Apr 7;21:666. doi: 10.1186/s12889-021-10695-y (PMC8028711; doi:10.1186/s12889-021-10695-y)
Supplement: Supplementary file 1 — Additional file 1 Appendix A Data collection instruments. The data collection tool contains the questionnaire and the guidelines for the focus group discussions (FGD) [file 12889_2021_10695_MOESM1_ESM.docx]

**APPENDIX A: DATA COLLECTION TOOLS**

YOUTH REPRODUCTIVE HEALTH SURVEY INSTRUMENT (ENGLISH)

| **Data collector code:** |  |
| --- | --- |
| **Respondent number:** |  |
| **Location code:** |  |
| **Date (day/month/year):** |  |
| **Respondent’s Sex** | - Male - Female |

**Demographics**

1. How old are you? _________years
2. What is the highest level of school that you have completed?

- Primary
- Secondary
- Tertiary
- University
- None

1. Are you currently attending school or university?

- Yes
- No

1. What is your relationship status?

- Married
- Unmarried, in a relationship
- Single
- Divorced
- Widow/Widower

**Family Planning Awareness**

1. Have you ever heard of the term “family planning*”* ***(If yes, go to question 6. If no, go to question 9)***

- Yes
- No

1. Where did you first hear this term?

- School
- Health facility
- VHT (Village Health Team)
- Radio or TV or newspapers
- Friends
- Parents/family
- Community group
- Other _________________

1. Have you ever received education or counseling on family planning or using family planning methods? ***(If yes, go to the next question, if no, go to question 9)***

- Yes
- No

1. Where did you receive this education or counseling about family planning?

- Hospital
- Community Clinic
- VHT (Village Health Team)
- Other _________________

**Sexual and Contraceptive Use History**

1. Have you ever had sexual intercourse?

- Yes ***(continue to question 10)***
- No ***(skip to question 17)***

1. How old were you when you first had sex?

Age in years: _________years

1. Are you currently having sex?

- Yes
- No

1. Do you have any children?

- Yes ***(continue to question 13)***
- No ***(skip to question 14)***

1. How many children do you have? *(if none, write “0” or “none”)* __________
2. Have you ever used a family planning method?

- Yes ***(continue to question 16)***
- *No* ***(skip to question 17)***

1. If yes, what family planning methods have you tried? ***(Please note all that you have tried) (After answering, please skip to question 17)***

- Condom
- Pills
- Injectable
- Implant
- IUD
- Tubal ligation
- Vasectomy
- Withdrawal
- Lactational amenorrhea (breastfeeding)
- Fertility awareness
- Other:___________________________

1. If you have **never**used a family planning method, what is your reason? ***(Please note all that apply)***

- I want to have children now
- Contraceptives cost too much
- My partner would not agree to use one
- I do not know where to get family planning services
- I worry about side effects
- It is too inconvenient to travel to get it
- I would not feel comfortable asking for it
- There are few places willing to give a family planning method to an adolescent
- Other:___________________________

1. Do you know where to get a family planning method such as condoms, pills, an IUD, or an implant if you wanted to obtain them for yourself?

- Yes ***(continue to question 18)***
- No ***(skip to question 19)***

1. Where would you go to receive these services?

- Hospital
- Health Center
- VHT (Village Health Team)
- Other _________________

1. If you ever had a question about family planning, where would you go for help? *(****Please note all that apply)***

- Friend
- Partner
- Internet
- VHT (Village Health Team)
- Teacher
- Parent/Relative
- Health Professional
- I don’t know where to go for help
- Other: _________________

1. Did you ever want to get a family planning method but someone told you not to?

- Yes ***(continue to question 21)***
- No (***skip to question 22)***

1. Who told you not to get family planning? ***(Note all that apply)***

- Friend
- Partner
- VHT (Village Health Team)
- Teacher
- Parent/Relative
- Health Professional
- I didn’t feel like I could talk to anyone about contraception
- Other: ______________________________________________________________________________________________________________________________________________________

1. Please tell me whether you agree or disagree with this statement, *“Adolescents should be able to get family planning services without being stigmatized by their family or health care providers”*

- Agree
- Disagree
- Neither agree nor disagree

**Awareness and Comfort with Village Health Teams**

*I am now going to ask you some questions about village health teams (VHT). Village health teams are members of the community who are trained to give health education, advice, and services in a village or town.*

1. Have you ever seen a village health team (VHT) working in this parish?

- Yes ***(continue to question 24)***
- No ***(skip to question 25)***

1. If yes, have you ever talked with a VHT in your community about health matters?

- Yes
- No

1. Do you think that VHTs are able to give family planning methods to young unmarried men and women for free?

- Yes
- No

1. How comfortable would you feel talking to a VHT about your health?

- Very comfortable
- Comfortable
- Neither comfortable or uncomfortable
- Somewhat uncomfortable
- Very uncomfortable

1. How comfortable would you feel discussing your family planning methods with a VHT?
   - Very comfortable ***(skip to question 29)***
   - Comfortable ***(skip to question 29)***
   - Neither comfortable or uncomfortable
   - Somewhat uncomfortable
   - Very uncomfortable
2. Which of the following are reasons why you might not feel comfortable talking to a VHT about family planning (check all that apply) :

- I would be afraid the VHT would share our conversation with my parents
- There would be no privacy when talking to VHTs
- I would not want to reveal that I am having sex
- I would feel shy talking about sex
- I would be afraid that the VHT is judging me
- I would be afraid that the VHT would not treat me well
- Other: ______________________________________________________________________________________________________________________________________________________________________________________________________________________________________________________

1. If you were deciding on whether or not to talk to a VHT about family planning, how important would it be that the VHT who is close to your age?

- Very important
- Important
- Neither important or unimportant
- Unimportant
- Not at all important

1. If you were deciding on whether or not to talk to a VHT about family planning, how important would it be that the VHT is the same gender as you?

- Very important
- Important
- Neither important or unimportant
- Unimportant
- Not at all important

1. If you wanted to get a family planning method, which of the following is the most important reason for asking for services from a VHT or a health center?

- Not needing parents’ permission
- Having the health facility or VHT be nearby
- High level of privacy
- Low cost or free services
- Friendly and respectful services
- Other: ________________________________________________________________________________________________________________________________________________________________________________________________________________________________________________________________________________________________________________________________________

1. What would be the second most important motivation?

- Not needing parents’ permission
- Nearby health facility or VHT
- High level of privacy
- Low cost or free services
- Friendly and respectful services
- Other: ________________________________________________________________________________________________________________________________________________________________________________________________________________________________________________________________________________________________________________________________________

Focus Group Discussion Guide

Abstracted from Chandler, C.I.R., and Reynolds, J. (2013) ACT Consortium Guidance: Qualitative Methods Protocol Template with example Tools and SOPs. Available at [www.actconsortium.org/qualitativemethodsguidance](http://www.actconsortium.org/qualitativemethodsguidance) and from Lundgren, R. Research Protocols To Study Sexual And Reproductive Health Of Male Adolescents And Young Adults In Latin America, Division of Health Promotion and Protection Family Health and Population Program, PAHO

| **FGD IDNO** \|__\|__\|__\|__\| **Facilitator Initials** \|__\|__\|__\| **Note-taker Initials** \|__\|__\|__\| **Audio file**: \|__\|__\|__\|  **Date** \|__\|__/__\|__/__\|__\|  **Introduction**  I am ______________________________ from ______________________ (Facilitator)  I am ______________________________ from ______________________ (note-taker)   - Ask group to introduce themselves using a nickname [petname] - Capture demographic details – using first name for discussion - Explain general purpose of the study: - *For FGD:* To understand the barriers youth face in accessing community based family planning services - *For overall study:* To see if there is any way for us to improve the delivery of family planning services for youth in Nakaseke - Aims of the discussion and expected duration (1 hour) - Why the participants’ cooperation is important - What will happen with the collected information and how the participant/target group will benefit - Tell group your ground rules and ask the group to define their own, for example: - Only one person talks at a time. - It is important for us to hear everyone’s ideas and opinions. There are no right or wrong answers to questions – just ideas, experiences and opinions, which are all valuable. - It is important for us to hear all sides of an issue – the positive and the negative. - Confidentiality is assured. Anything you say here will be kept private and confidential. We’ll never mention your name - outside of this room. If you prefer not to answer any particular question, that’s fine. If you need to leave at any time, that is okay. “What is shared in the room stays in the room.” - Any questions? - Check position and functioning of tape recorder - Check for everyone’s consent to participate and be recorded - Refreshments will be served after the discussion   **Now I am going to introduce some topics one at a time about your experiences and I hope you can discuss them together. Remember, there are no right or wrong answers to the questions I’ll be asking you. Please feel free to  answer exactly as you feel.** |
| --- |

[Note: these are preliminary questions. It is expected that these questions will change to take into account survey findings]

| **Domain** | **Topic and Probes** |
| --- | --- |
| Sources of information | 1. Where do young people like you learn about their body and how to take care of it? Where do they learn about sex?    1. *Probe for mass media, parents, friends, teachers, religious figures.*    2. *Probe for type of information they receive and their opinion of the information* 2. If there were a place to learn about sex and your body, what would you like to know? Who would like to talk to? |
| Family planning use & barriers | 1. How often do you think most young people like you use a family planning method? Why don’t they use one every time?    1. Why?    2. What methods do they use? Why? 2. Do you think it is easy or hard to get a family planning method?    1. Why?    2. What are the barriers to getting a family planning method |
| Family planning services | 1. If you needed a family planning method, where would you go? Why? 2. Most family planning services are designed for adults. I want you to pretend that this group has been asked to design the ideal health services for young people. What would they be like? (Note comments on a flip chart sheet)    1. Probe for:       1. services provided       2. hours and scheduling procedures       3. physical structure       4. separate from women/adults       5. privacy/confidentiality       6. prices       7. provider characteristics    2. Probe: Would these services be different than those for adults? In what ways? |
| VHTs | 1. Have any of you ever talked to a VHT about your health? Do you have VHTs in your community? 2. How do you feel about receiving family planning services from a VHT? 3. What are your concerns about seeking family planning help from a VHT?    1. Probe for concerns age, gender, confidentiality, expertise |
| Closing  We are now approaching the end of our discussion. Is there anything else anyone would like to add about family planning that we have not talked about?   - Summarise - Thank participants - Collect participant demographic details | |

Focus Group Participants Demographic Background Sheet

| **FGD IDNO:**  \|__\|__\|__\|__\| **Facilitator Initials:**  \|__\|__\|__\| **Note-taker Initials:**  \|__\|__\|__\|  **Audio file**: \|__\|__\|__\|  **Date** \|__\|__\|/\|__\|__\|/\|__\|__\| | |
| --- | --- |
| **Questions** | **Answers** |
| 1. How old are you? | _______ years old |
| 1. What was your level of education? | - None - Some primary - Completed primary - Some secondary - Completed Secondary - Post-Secondary |
| 1. How many children do you have? |  |
